# Natural product-based bioactive agents in combination attenuate neuroinflammation in a tri-culture model

**DOI:** 10.3389/fphar.2023.1135934

**Published:** 2023-02-15

**Authors:** Yang Liu, Dennis Chang, Tianqing Liu, Xian Zhou

**Affiliations:** ^1^ NICM Health Research Institute, Western Sydney University, Westmead, NSW, Australia; ^2^ School of Science, Western Sydney University, Penrith, NSW, Australia

**Keywords:** neuroinflammantion, herbal compound formulation, tri-culture, BBB, neuroprotection

## Abstract

**Introduction:** Neuroinflammation is an important pathological event contributing to the onset and progression of neurodegenerative diseases. The hyperactivation of microglia triggers the release of excessive proinflammatory mediators that lead to the leaky blood-brain barrier and impaired neuronal survival. Andrographolide (AN), baicalein (BA) and 6-shogaol (6-SG) possess anti-neuroinflammatory properties through diverse mechanisms of action. The present study aims to investigate the effects of the pair-combinations of these bioactive compounds in attenuating neuroinflammation.

**Methods:** A tri-culture model with microglial N11 cells, microvascular endothelial MVEC(B3) cells, and neuroblastoma N2A cells was established in a transwell system. AN, BA and 6-SG used alone (25 µM) or in pair-wised combinations (12.5 + 12.5 µM) were subjected to the tri-culture system. Upon the stimulation of lipopolysaccharides (LPS) at 1 μg/mL, tumor necrosis factor-alpha (TNF-α) and interleukin 6 (IL-6) levels were determined by ELISA assays. Immunofluorescence staining was applied to investigate the nuclear translocation of nuclear factor kappa B p65 (NF-κB p65) on N11 cells, expressions of protein zonula occludens-1 (ZO-1) on MVEC cells and phosphorylated tau (p-tau) on N2A cells, respectively. The endothelial barrier permeability of MVEC cells was assessed by the Evans blue dye, and the resistance from the endothelial barrier was measured by transepithelial/endothelial electrical resistance (TEER) value. Neuronal survival of N2A cells was determined by Alamar blue and MTT assays.

**Results:** Combinations of AN-SG and BA-SG synergistically lowered the TNF and IL-6 levels in LPS-induced N11 cells. Remarkably, the combined anti-neuroinflammatory effects of AN-SG and BA-SG remained significantly greater compared to their individual components at the same concentration level. The molecular mechanism of the attenuated neuroinflammation was likely to be mediated by downregulation of NF-κB p65 translocation (*p* < 0.0001 vs. LPS stimulation) in N11 cells. In the MVEC cells, both AN-SG and BA-SG restored TEER values, ZO-1 expression and reduced permeability. Furthermore, AN-SG and BA-SG significantly improved neuronal survival and reduced expressions of p-tau on N2A cells.

**Discussion:** The AN-SG and BA-SG combinations showed greater anti-neuroinflammatory potential than those used alone in mono- and tri-cultured N11 cells, thereby further protecting endothelial tight junction and neuronal survival. Taken together, AN-SG and BA-SG may provide improved anti-neuroinflammatory and neuroprotective activities.

## 1 Introduction

Neurodegenerative diseases are a group of conditions, including motor neuron disease, Alzheimer’s disease, Parkinson’s disease and Huntington’s disease, characterized by the progressive loss of structure or function of neurons (Fumia et al., 2022). According to World Health Organization, there are more than 55 million people suffering from dementia worldwide (World Health Organization, 2022). Due to the rapid growth of the aging population, neurodegenerative diseases are predicted to be the top disease burden in 2050 (Nichols et al., 2022). The conventional treatments for neurodegenerative diseases are largely symptomatic and present very limited effectiveness in curing or preventing the disease. Thus, it is imperative to explore effective therapeutic options (Yang et al., 2021a).

Neuroinflammation is inflammation occurring in the central nervous system (CNS). It is a pathological event that is related to the onset of neuronal damage and contribute to the progression of neurodegeneration and cognitive impairment (Guzman-Martinez et al., 2019). Neuroinflammation is primarily initiated by the activation of glial cells (mainly microglia) and consequential expression of proinflammatory mediators (Adriani et al., 2017; Bhowmick et al., 2019). Upon the activation, microglia secrete proinflammatory mediators/cytokines such as nitric oxide (NO), tumor necrosis factor (TNF)-α and interleukin (IL)-6 (Ha et al., 2012), of which the overproduction in a chronic condition can contribute to the impairment of the blood-brain barrier (BBB) and result in higher permeability of toxins (Takata et al., 2021). The breakdown of the BBB allows further immune cell recruitment, which then attack the myelin around nerves and result in nerve damage leading to escalated neuroinflammation (Kempuraj et al., 2016). In addition, emerging evidence has shown that the crosstalks among various groups of glial cells, and between microglia and neuron within the neurovascular unit (NVU) are important in the pathogenesis of neuroimmunomodulation in CNS (De la Fuente, 2008; Fernández et al., 2008; Maccioni et al., 2009; Maccioni et al., 2018). Although the understanding of how microglia-neuron cellular interaction occurs in CNS neuroinflammation remains limited, a recent study has shown that microglia-mediated neuroinflammation is linked with neuronal injury and phosphorylation of tau and tauopathies (Guzman-Martinez et al., 2019). The physiological function of the tau protein can be altered by the chronic stimulation of glial cells, which leads to the activation of enzymes that phosphorylate tau, then reduced neuronal capacity (Wyss-Coray and Mucke, 2002). All these interactions and positive feedback loop between the tau pathology and the activation of glial cells cause continuous neuroinflammatory cycles eventually leading to neurodegeneration (Morales et al., 2014).

Robust *in vitro* and *in vivo* models that can measure the multifaceted interactions are key to the understanding of complex pathophysiology of neuroinflammation and to the development of therapeutic interventions. Research has been devoted to establishing co- and tri-culture models as a practical *in vitro* tool for screening brain-targeted drug candidates before animal testing. For example, Park et al. (2018) established a three dimensional human tri-culture system (neuron, astrocyte, and microglia) modeling neuroinflammation, which demonstrated that the induced microglial recruitment released pro-inflammatory cytokines and chemokines, and resulted in the death of neurons and astrocytes . Our previous study established a tri-culture model (microglial, endothelial and neuronal cells) to simulate the NVU environment under neuroinflammation. It was observed that the activated microglia directly provoked the damage of the endothelial tight junction and triggered neuronal loss (Zheng et al., 2021).

The current treatment of neurodegenerative diseases largely focuses on symptomatic relief. Cholinesterase enzyme inhibitors such as galantamine, donepezil, rivastigmine, and N-methyl d-aspartate antagonists (i.e., memantine) have shown to improve memory, thinking, judgment and other thought processes. However, none of these treatments could prevent or stop the progression of the diseases (Van Bulck et al., 2019). Moreover, the etiologic and underlying pathophysiology of neurodegenerative diseases are variable and includes different factors (Leroi et al., 2006), and thus the multi-component and multi-target approach (as opposed to mono-component and mono-target therapy) may offer a better solution to combat the diseases (Liu et al., 2017). Herbal medicines have been extensively explored in recent years regarding their biological activities and potential therapeutic benefits for neurodegenerative disorders (Mohd Sairazi and Sirajudeen, 2020) (Mecocci et al., 2014; Makkar et al., 2020). Emerging evidence has shown that some herbal medicines and their bioactive components used in combination can illustrate synergistic and multi-target effects (Liebner et al., 2018; Choi et al., 2011; Lin, 2011; Calfio et al., 2020). Yang et al. (2021b) demonstrated that a herbal combination of *Aconitum carmichaelii* Debx. (*Fuzi*) and *Zingiber officinale* Rosc. (*Ganjiang*) exhibited a multi-target behavior against neuroinflammation as evidenced by reduced productions of IL-6, TNF-α, reactive oxygen species, NO, and prostaglandins E2 in microglia BV2 cells which were likely to be attributed to the interactions of their bioactive components.

Andrographolide (AN), a major bioactive component of *Andrographis paniculata*, exhibits a wide range of biological activities, including anti-inflammatory, antioxidant, anti-neuroinflammatory, and neuroprotective effects (Zhang et al., 2021). The mechanisms underlying its anti-neuroinflammatory effect were found to be associated with the inhibition of nuclear factor kappa B p65 (NF-κB) signaling and the activation of NLR family pyrin domain containing 3 (Li et al., 2018). Baicalein (BA), a flavone subclass of flavonoids, is a major bioactive constituent in the roots of *Scutellaria baicalensis* (Yang et al., 2019). This compound has been shown to possess various biological characteristics, including anti-bacterial, anti-hypertensive, and anti-neuroinflammatory effects (Zhang et al., 2017). BA was shown to reduce the levels of a broad range of pro-inflammatory cytokines, such as IL-1, TNF-α, and IL-6, *via* the NF-κB signaling inhibition (Zhang et al., 2017). 6-shogaol (6-SG), a pungent component derived from ginger *Zingiber officinale* Rosc., is another promising bioactive agent that exhibits neuroprotective and anti-inflammatory properties (Park et al., 2013). 6-SG was shown to significantly suppress TNF-α and NO levels through downregulation of cyclooxygenase (COX-2), p38 mitogen-activated protein kinase (MAPK), and NF-κB signaling pathways in the lipopolysaccharides (LPS)-induced microglia BV2 cells and a neuroinflammatory mouse model (Ha et al., 2012). It appears that AN, BA and 6-SG all possess anti-neuroinflammatory properties, with the NF-κB signaling as the common pathway. However, it is plausible that their combined use can generate a multi-target behavior against neuroinflammation in NVU attributed to their versatile pharmacological actions.

This study aims to explore the anti-neuroinflammatory effects of AN-SG and BA-SG combinations on protecting neurons and endothelial tight junctions in an LPS-induced neuroinflammation tri-culture model.

## 2 Materials and methods

### 2.1 Cell culture

#### 2.1.1 Cell lines

The mouse brain microvascular endothelial cell line MVEC(B3) was obtained from Dr Jia Li, Macquarie University. Mouse microglia N-11 (N11) and mouse neuroblastoma Neuro 2A cell lines (N2A) were kindly donated from Professor Gerald Muench, School of Medicine, Western Sydney University. They were cultured in complete Dulbecco’s modified Eagle medium (DMEM, Lonza, Australia) supplemented with 10% foetal bovine serum (FBS, Thermo Fisher Scientific, Australia) and 1% penicillin.

#### 2.1.2 Cell culture for the single cell line

N11 cells, MVEC cells and N2A cells were incubated at 37°C, 5% CO_2_ in 95% air in a vented flask T75 cm^2^ (Sigma-Aldrich, Australia), respectively. Cultured cells up to passage 35 with approximately 90% confluency were digested with 0.25% trypsin (Thermo Fisher Scientific, Australia) to perform experiments.

#### 2.1.3 Mono-culture and tri-culture system

For mono-culture, N11 cells (1.0 × 10^6^ cells/mL) were seeded in 96-well plates with DMEM containing 10% FBS. A tri-cultured neuroinflammation model was established using transwell polycarbonate membrane cell culture inserts in a 24-well plate (Corning®Costar®Transwell^®^ Cell culture inserts, Sigma, Australia) with slight modification (Zheng, et al., 2021). The transwell with insert membrane was placed upside down and coated with 1% polylysine (Sigma, United States) for 1 h. Then 200 µL of N11 cells (2.0 × 10^6^ cells/mL) were seeded on the top of the transwell with DMEM containing 10% FBS and kept upside down in the incubator to ensure N11 cells adhered to the top of the transwell by the surface tension. In parallel, 500 µL of N2A cells (1.0 × 10^6^ cells/mL) were seeded in a fresh 24-well plate with DMEM containing 10% FBS. After 4 h, the transwell with N11 cells was inverted and placed within a 24-well plate immersed with 375 µL DMEM containing 10% FBS in each well, and the MVEC cells (1.0 × 10^6^ cells/mL) were seeded with 250 µL DMEM containing 10% FBS in the same insert of N11 cells.

### 2.2 Drug preparation and lipopolysaccharides-induced neuroinflammation

The natural compounds, AN, BA and 6-SG (purity >98%), were purchased from Chengdu Biopurify (China). The identity and purity were confirmed by high-performance liquid chromatography (Supplementary material 1). Each compound was dissolved in dimethyl sulfoxide (DMSO) at a concentration of 100 mM. They were serially diluted with DMEM serum-free media before adding to the cells. After the three cell lines were cultured together in the transwell system for 24 h, the system was treated with individual AN, 6-SG, and BA at 25 μM, combined AN-SG [AN (12.5 µM) +6-SG (12.5 µM), the total concentration of 7.83 μg/mL], and BA-SG [BA (12.5 µM) + 6-SG (12.5 µM), total concentration of 6.83 μg/mL] or media with vehicle control (0.1% DMSO) for 1 h before the activation of LPS, 1 μg/mL. The cells and cell supernatant were then subjected to bioassays after 24 h.

### 2.3 Measurement of nitrite (NO_2_^−^) production using the griess reaction

After 24 h of LPS stimulation, the supernatants in the upper and lower compartment (90 µL) in the tri-culture system were collected for the measurement of nitrite (NO_2_^−^) level by mixing with an equal amount of the Griess reaction (1% sulfanilamide in 5% phosphoric acid and N-(1-naphthyl)-ethylene diamine dihydrochloride) (Tsikas, 2007; Yoon, et al., 2010). The measurement of nitrate/nitrite concentration or total nitrate and nitrite concentration (NOx) is routinely used as an index of NO production (Moncada et al., 1991; Sun et al., 2003). Griess assay has been popularly used to measure the nitric oxide (NO) level (Bohl and West, 2000; Saha et al., 2004; Schmölz et al., 2017). Nitrite production was determined by measuring the optical density at 540 nm using a microplate reader (BMG Labtech Fluostar Optima, Mount Eliza, Victoria, Australia). The concentration of nitrite was determined using a standard curve generated with sodium nitrite (NaNO_2_). The rest of the cell supernatant was subjected to TNF-α and IL-6 ELISA assay.

### 2.4 TNF-α and IL-6 ELISA assay

The levels of IL-6 and TNF-α in the supernatant from the mono-cultured N11 cells, the upper and lower compartment of the tri-culture system were measured using commercial ELISA kits (murine IL-6 ELISA kit, cat. No. 431304, Biolegend; murine TNF-α ELISA kit, cat. No. 900- K45, Biogems) according to manufacturers’ instructions. The absorbance was measured at 410 nm with a microplate reader (BMG Labtech Fluostar Optima, Mount Eliza, Victoria, Australia). The concentrations of IL-6 and TNF-α were calculated using standard curves.

### 2.5 Transendothelial electrical resistance values

The transendothelial electrical resistance (TEER) values in the tri-culture 24-well systems were measured by an epithelial volt/ohm resistance meter (ERS-2, cat. no. MERS00001; Merck) according to the manufacturer’s instruction. The background TEER value was measured in the blank well with medium only. The final results were calculated by TEER in each group subtracted from the background TEER values. The values are shown as Ω × cm^2^.

### 2.6 Evans blue permeability test

After the various treatments for 24 h in the tri-culture system, the medium in the lower compartment was replaced with 0.5 mL phosphate-buffered saline (PBS), and the upper compartment was filled with 0.2 mL of 0.25% Evans blue (Sigma, Australia). After 0.5 h’s incubation, the solution in the lower compartment was tested for absorbance at 450 nm using a microplate reader (BMG Labtech Fluostar Optima, Mount Eliza, Victoria, Australia).

### 2.7 Alamar blue and MTT assays

Cell viability of N2A cells in the tri-culture system was evaluated 24 h after the LPS stimulation. After removing the supernatants, the N2A cells were incubated with 100 µL of Alamar Blue (0.01 mg/mL resazurin) (Schober, et al., 2021). The plate was then incubated for another 2 h in a humidified incubator at 37°C. The optical density of each well was measured from excitation of 545 nm and emission of 595 nm using a microplate reader (BMG Labtech Fluostar Optima, Mount Eliza, Victoria, Australia).

The N2A cells were also co-incubated with MTT solution (0.5 mg/mL in PBS) 24 h after the LPS stimulation for 2 h at 37°C with 5% CO_2_. DMSO was then added to dissolve the insoluble formazan crystal. The absorbance was measured at 510 nm using a microplate reader. The density of formazan formed in control (medium with the vehicle) cells was taken as 100% of cell viability. The cell viability of the measured sample was determined using the equation: Cell viability % = absorbance of treated cells/absorbance of cells and medium only (blank control) * 100%

### 2.8 Immunofluorescent staining

The N2A cells were seeded on a 1 cm cover slide in the 24-well. The tri-culture system was established and treated overnight using the same protocol as described above. Each cell line was washed with cold PBS and fixed with 4% paraformaldehyde for 20 min at room temperature. Triton-X 100 (0.1%) was added to cells for 20 min and followed by blocking with 3% Bovine Serum Albumin (BSA, Scientifix, Australia) for 1 h. Primary antibodies were then added to the system, including zonula occludens-1 (ZO-1; 1:100, cat no. 13663S), phosphorylated tau (p-tau; 1:200, cat no. 49561) or NF-κB p65 (p65; 1:100, cat no.8242), all purchased from Cell Signaling Technologies, United States. The cells were then washed with PBS three times and each cell line was then stained with mixed secondary antibodies, including donkey anti-mouse IgG Alexa Fluor 488 (green; 1: 1,000, cat no. A32766), donkey anti-goat IgG Alexa Fluor 680 (yellow; 1: 250, cat no. A32680) and donkey anti-rabbit IgG Alexa Fluor 594 (red; 1: 1,000, cat no. A32754), all purchased from Thermo Fisher Scientific, Australia. The insert membrane was cut for the collection of either the N11 or the MVEC cells. Cotton was used to swap the other side of the membrane of the cell line and then put on the new slide. Finally, the anti-fade mounting medium with DAPI (Sigma, Australia) was added to the cells and subjected to immunofluorescent imaging using an Inverted Leica TCS SP5 laser scanning confocal microscope (School of Medicine, Western Sydney University, Australia).

### 2.9 Statistical analysis

Statistical analysis was conducted using GraphPad Prism 9.0 software (GraphPad Software Inc., United States). The data was shown as mean ± standard error of the mean (SEM) from at least three individual experiments. The relative half maximal inhibitory concentration (IC_50_) values were determined from the constructed dose-response curves. The responses generated by nitrite, TNF and IL-6 assays were normalised to 0%–100%, where the averaged responses of blank control and LPS were normalised to 0% and 100% respectively. The IC_50_ value was then determined by the concentration that corresponds to 50% of the effect level. The statistical comparison between groups was conducted by one-way analysis of variance (ANOVA) with Tukey test, and *p* < 0.05 was considered statistically significant. The interaction of the substances in the ELISA experiment was ascertained using the combination index (CI) model. The data obtained from the dose-response curves in reducing IL-6 and TNF-α was entered into the Compusyn software 2.0 (ComboSyn, Inc., United States), which produced the isobologram figure, CI values, and CI-fraction affected (Fa, here refereed to IL-6/TNF-α inhibition) curve (El Hassouni et al., 2019).

## 3 Results

### 3.1 Enhanced anti-neuroinflammatory activities of AN-SG and BA-SG

#### 3.1.1 Restored cell viability by AN-SG and BA-SG

**FIGURE 1:**
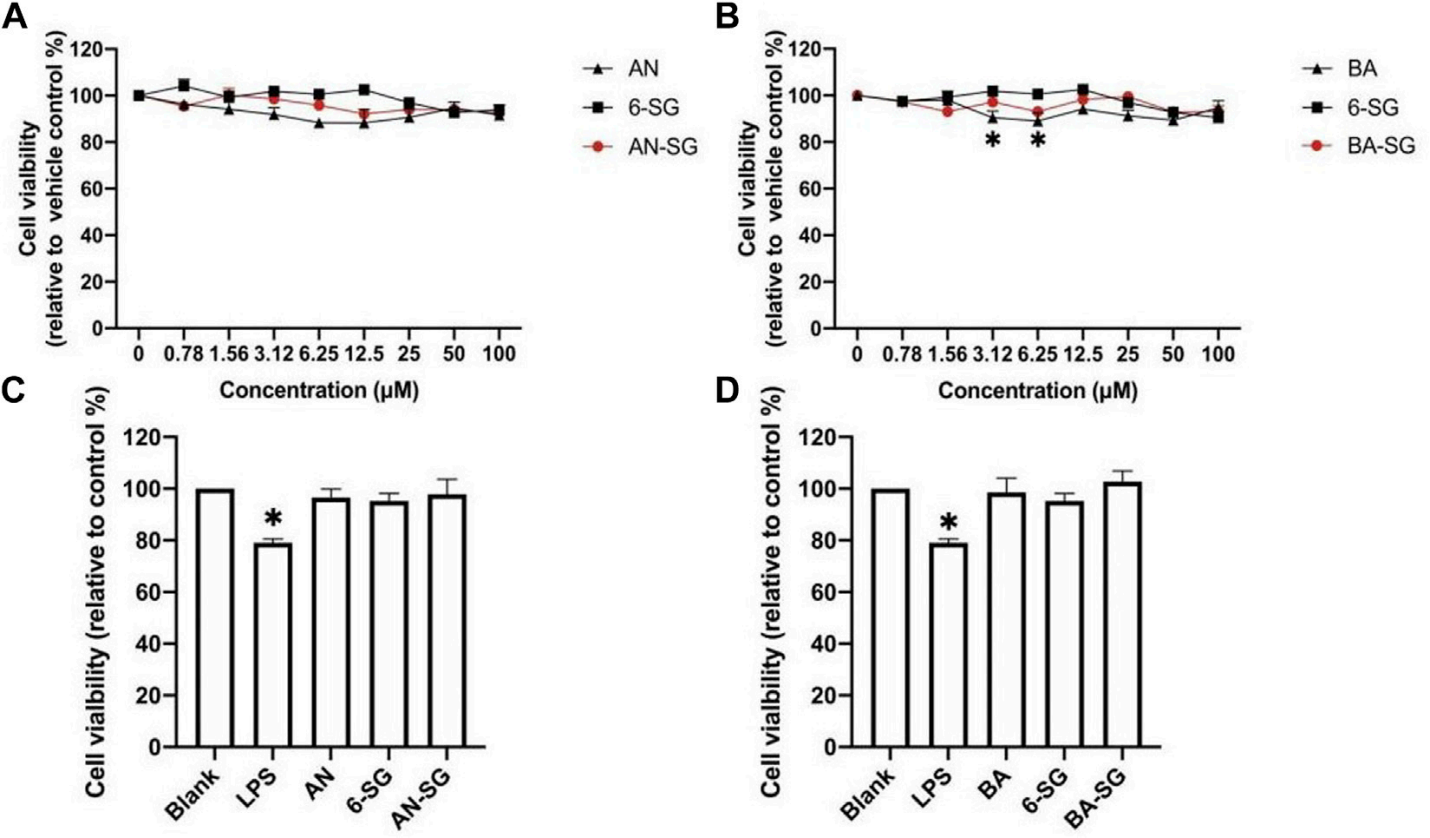
Cell viability of N11 cells in the mono-culture treated by AN, BA, 6-SG and their combinations, AN-SG and BA-SG. **(A)** The dose-response curve of the cell viability treated by AN, 6-SG and AN-SG at 0.78–100 µM in N11 cells. **(B)** The dose-response curve of the cell viability treated by BA, 6-SG and BA-SG at 0.78–100 µM in N11 cells. **(C)** Cell viability of N11 treated by AN, 6-SG and AN-SG at 25 µM. **(D)** Cell viability of N11 treated by BA, 6-SG and BA-SG at 25 µM *p < 0.05 vs. Blank (cells with vehicle control) analysed by one-way ANOVA with Tukey test. Data are shown as mean ± SEM (*n* = 3).

Firstly, the cell viability of N11 cells in the mono-culture and tri-culture system treated by the individual and combined bioactive was examined. As shown in Figures 1A, B, AN and 6-SG (0.78–100 µM) did not exhibit any obvious cytotoxicity, which were all above 88.27% compared with the vehicle control (blank). Particularly, 6-SG showed the highest cell viability throughout the tested concentrations, which ranged from 92.72% ± 2.17% to 104.13% ± 4.85%. The treatments of BA and AN appeared to slightly affect the cell viability, which ranged from 89.07% ± 5.93% to 97.92% ± 0.85%, and 88.27% ± 8.34% to 96.25% ± 0.34%, respectively. BA at 3.125 and 6.25 µM showed significantly impaired cell viability compared to the blank control (*p <* 0.05). Interestingly, combinations of AN-SG and BA-SG did not show any obvious cytotoxicity. AN-SG seemed to lower reduction in cell viability compared to that of AN alone, and a similar trend was shown in BA-SG compared to that of BA alone.

In particular, the cell viability of mono-cultured N11 cells treated by AN, BA, 6-SG, AN-SG, and BA-SG at 25 µM were tested (Figures 1C, D). It was obvious that LPS stimulation significantly affected the cell viability of N11 cells compared to the vehicle control (79.00% ± 2.64% vs. blank 100%, *p* < 0.05). All the treatments did not exhibit any cytotoxicity at 25 µM compared with the blank, with cell viability all above 95.28% ± 8.59%. Particularly, AN-SG 25 µM (12.5 + 12.5 µM, 7.83 μg/mL), BA-SG 25 µM (12.5 + 12.5 µM, 6.83 μg/mL) maintained the cell viability at 97.89% ± 11.48% and 102.76% ± 7.14%.

#### 3.1.2 Synergistic inhibition of IL-6 and TNF-α productions by AN-SG and BA-SG on mono-cultured N11 cells

**FIGURE 2:**
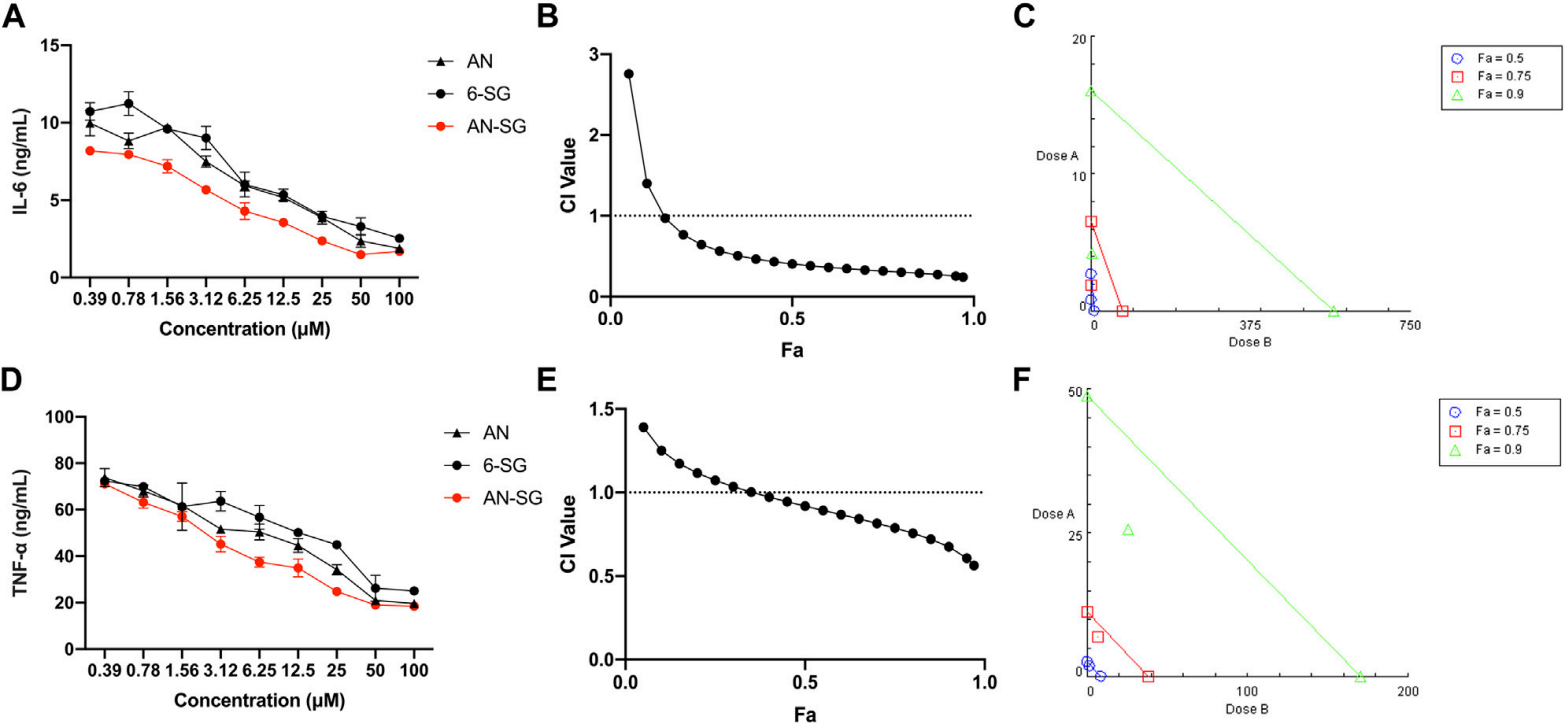
The AN-SG combination exhibited synergistic effects on inhibition of LPS-induced IL-6 and TNF-α productions in mono-cultured N11 cells. AN, 6-SG and AN-SG dose-dependently inhibited LPS-induced IL-6 **(A)** and TNF-α **(D)** in N11 cells (*n* = 3). The synergistic IL-6 **(B)** and TNF-α **(E)** inhibitory effects of AN-SG were determined by the CI-Fa curves. CI values represent the interaction in AN-SG, with CI < 1, CI = 1 and CI > 1 referring to synergy, addition and antagonism, respectively. Fa on the *X*-axis is defined as the fraction effect level, and herein it refers to the IL-6 and TNF-α inhibitory effect, respectively. Isobologram analysis of AN-SG in IL-6 **(C)** and TNF-α **(F)** inhibition when the default set of Fa values at 0.50, 0.75 and 0.90. Data are shown as mean ± SEM.

Next, the IL-6 and TNF-α inhibitory effects of AN-SG were tested in LPS-induced mono-cultured N11 cells compared with AN and 6-SG used alone (Figures 2A, D). LPS-stimulated cells demonstrated significantly increased amounts of IL-6 (12.29 ± 1.13 ng/mL vs. 2.06 ± 0.08 ng/mL, *p* < 0.0001) and TNF-α (78.52 ± 0.35 ng/mL vs. 14.25 ± 0.14 ng/mL, *p* < 0.0001) compared to that of the blank control. All the individual and combined treatments showed dose-dependent reductions of IL-6 and TNF-α compared to that of LPS (all *p* < 0.0001) on mono-cultured N11 cells. AN-SG and BA-SG (0.39–100 µM) appeared to be more potent than their individuals used alone at all tested concentrations. The IC_50_ value of AN-SG in inhibiting IL-6 was determined to be 1.18 ± 0.38 µM, which was significantly lower than that of AN (3.54 ± 1.19 µM, *p* < 0.01) or 6-SG (5.46 ± 1.41 µM, *p* < 0.01). Similarly, the IC_50_ value of AN-SG in inhibiting TNF-α was also the lowest (3.47 ± 0.64 µM) compared to that of AN (7.24 ± 1.90 µM, *p* < 0.05) or 6-SG (13.93 ± 6.37 µM, *p* < 0.001).

At 25 μM, the IL-6 production of AN-SG (2.51 ± 0.81 ng/mL) was significantly lower than that of the LPS stimulation (12.29 ± 1.13 ng/mL, *p* < 0.0001). In addition, IL-6 production by AN-SG was significantly lower than that of AN (4.14 ± 0.71 ng/mL, *p* < 0.01) or 6-SG (4.28 ± 0.77 ng/mL, *p* < 0.01). The TNF-α release of AN-SG (24.76 ± 1.41 ng/mL, *p* < 0.0001 vs. LPS) was also significantly lower than that of AN (34.22 ± 3.14 ng/mL, *p* < 0.05) or 6-SG (44.89 ± 2.63 ng/mL, *p* < 0.001) at 25 µM. Thus, this concentration was selected for AN, 6-SG and AN-SG to be tested in the tri-culture system.

The CI model was performed to evaluate whether the enhanced activity of the AN-SG combination was due to a synergistic interaction. In Figure 2B, the CI values ranged from 0.24 to 0.97 when the Fa was above 0.15 (15%–97% IL-6 inhibitory effect), suggesting a strong synergy of AN-SG combination in inhibiting IL-6. The isobologram (Figure 2C) also suggested a synergistic interaction between AN and 6-SG in reducing LPS-stimulated IL-6 with Fa values at 0.50, 0.75 and 0.9 (representing 50%, 75% and 90% of the IL-6 inhibition). Similarly, in Figure 2E, AN-SG synergistically inhibited TNF-α with CI values ranging from 0.56 to 0.97 when the Fa was above 0.4 (40%–97% TNF-α inhibitory effect). The results of the isobologram (Figure 2F) also supported this finding with Fa values at 0.50, 0.75 and 0.9.

**FIGURE 3:**
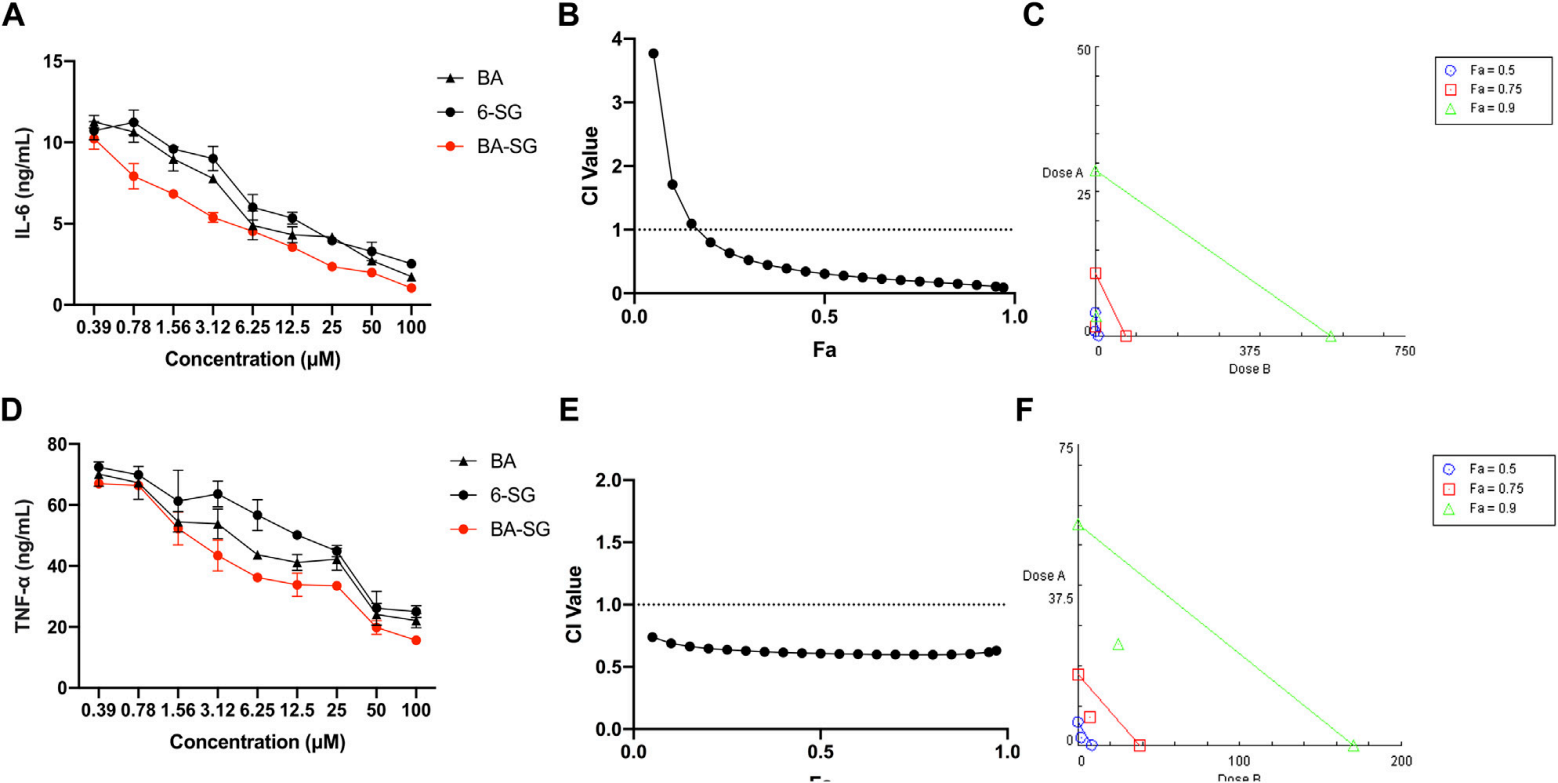
The BA-SG combination exhibited synergistic inhibitory effects on LPS-induced IL-6 and TNF-α productions in mono-cultured N11 cells. BA, 6-SG, and BA-SG dose-dependently inhibited LPS-induced IL-6 **(A)** and TNF-α **(D)** in N11 cells (*n* = 3). The synergistic IL-6 **(B)** and TNF-α **(E)** inhibitory effects of BA-SG were determined by the CI-Fa curves. CI values represent the interaction in BA-SG, with CI < 1, CI = 1 and CI > 1 referring to synergy, addition and antagonism, respectively. Fa on the *X*-axis is defined as the fraction effect level, and herein it refers to the IL-6 and TNF-α inhibitory effect, respectively. Isobologram analysis of BA-SG in IL-6 **(C)** and TNF-α **(F)** inhibition when the default set of Fa values at 0.50, 0.75, and 0.90. Data are shown as mean ± SEM.

The IL-6 and TNF-α inhibitory effects of BA-SG were examined in LPS-induced mono-cultured N11 cells against their individual components. Compared with LPS-stimulation, all the BA, 6-SG and BA-SG produced dose-dependent IL-6 and TNF-α inhibitory effects (Figures 3A, D). The IC_50_ value of BA-SG in inhibiting IL-6 was determined to be 1.44 ± 0.37 µM, which was significantly lower than that of BA (3.65 ± 0.90 µM, *p* < 0.05) or 6-SG (5.46 ± 1.41 µM, *p* < 0.01). Similarly, the IC_50_ value of BA-SG for the TNF-α inhibition was also the lowest (3.26 ± 1.09 µM) compared to that of BA (6.52 ± 2.93 µM, *p* < 0.05) or 6-SG (13.93 ± 6.37 µM, *p* < 0.01).

At 25 μM, BA-SG exhibited significant IL-6 inhibitory effects (2.47 ± 0.78 ng/mL, *p* < 0.0001) compared LPS stimulation only. In particular, the IL-6 production of BA-SG was significantly lower than that of BA (4.14 ± 0.63 ng/mL, *p* < 0.05) or 6-SG (4.28 ± 0.77 ng/mL, *p* < 0.05) alone. The TNF-α reduction by BA-SG was also significantly greater than that of BA (42.08 ± 3.62 ng/mL, *p* < 0.05) or 6-SG (44.89 ± 2.63 ng/mL, *p* < 0.05) alone at 25 µM. Thus, this concentration was selected for BA, 6-SG and BA-SG to be tested in the tri-culture system. CI and isobologram models (Figures 3B–F) demonstrated a synergistic effect of BA-SG in inhibiting IL-6 and TNF-α at the concentration range of 0.78–100 μM and 0.39–100 µM (CI < 1) were observed. The isobologram also supported the observed synergy of the BA-SG combination in reducing LPS-stimulated IL-6 (Figure 3C) and TNF-α (Figure 3F) when Fa values were at 0.50, 0.75, and 0.9.

#### 3.1.3 Enhanced inhibitory effects of AN-SG and BA-SG on nitrite, IL-6 and TNF-α productions in tri-culture system

**FIGURE 4:**
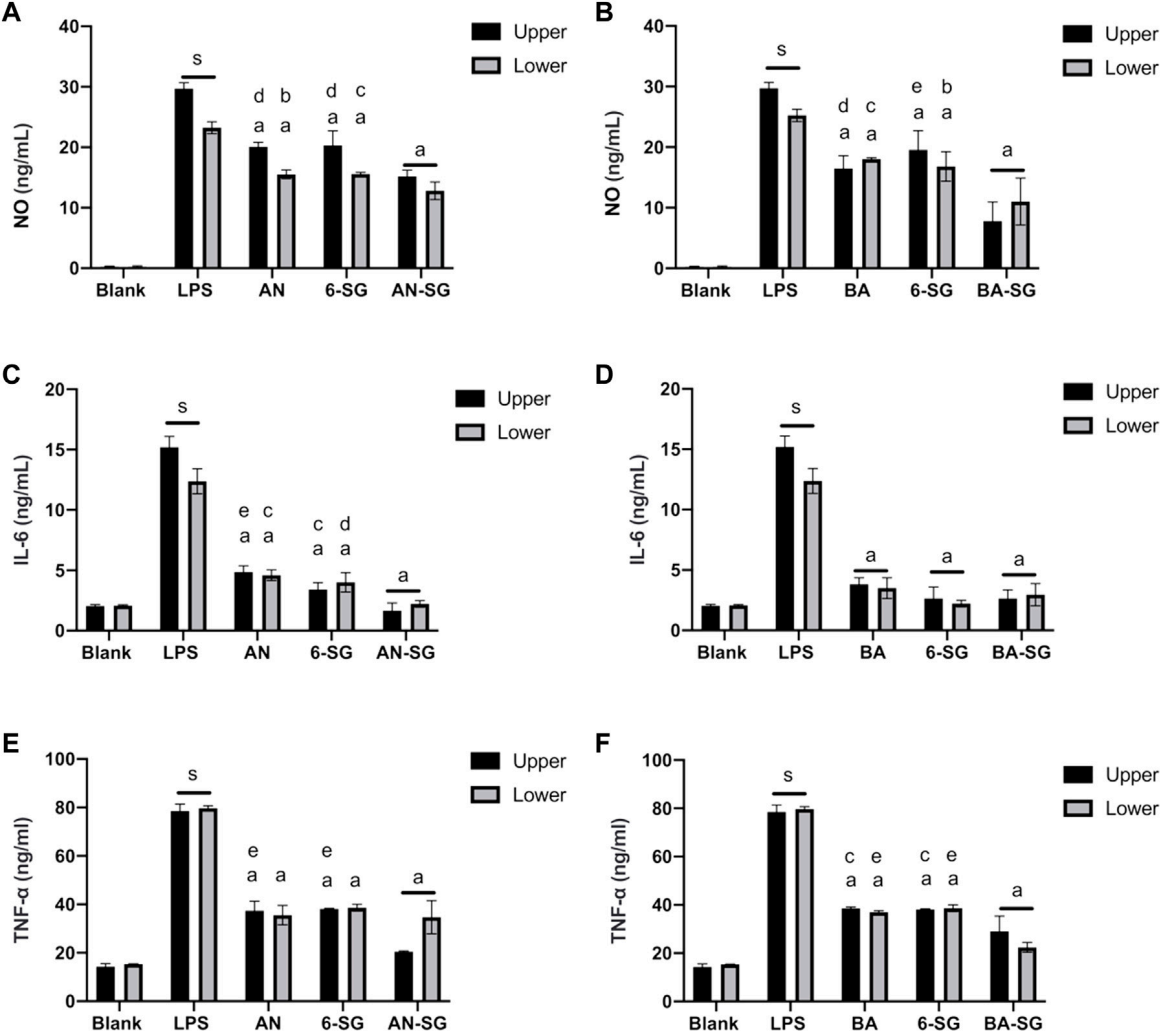
AN-SG and BA-SG generally exhibited greater effects in reducing nitrite, IL-6 and TNF-α productions than that of the individual component in the tri-culture system. AN-SG inhibited nitrite **(A)**, IL-6 **(C)**, and TNF-α **(E)** productions and BA-SG inhibited nitrite **(B)**, IL-6 **(D)** and TNF-α **(F)** productions in LPS-activated tri-culture model in both upper and lower compartments. Data are shown as mean ± SEM (*n* = 3). Figures generated by Graphpad prism 9.0. The statistical comparison between groups was conducted by one-way ANOVA analysis and Tukey test for group comparison. s: *p* < 0.0001 vs. Blank, a: *p* < 0.0001 vs. LPS, b: *p* < 0.05 vs. combinations, c: *p* < 0.01 vs. combinations, d: *p* < 0.001 vs. combinations, e: *p* < 0.0001 vs. combinations.

In the tri-culture model, nitrite, IL-6 and TNF-α inhibitory effects of AN-SG against LPS stimulation were compared to that of AN or 6-SG alone at 25 µM. As shown in Figure 4A, LPS generated an excessive amount of nitrite in the upper compartment at 29.71 ± 0.97 ng/mL (*p* < 0.0001 vs. blank control: 0.24 ± 0.13 ng/mL). The combination of AN-SG [AN (12.5 µM) + 6-SG (12.5 µM), total concentration of 7.83 μg/mL] significantly lowered the level of nitrite to 15.16 ± 1.06 ng/mL (*p* < 0.0001 vs. LPS stimulation) and the reduction was significantly greater than that of AN (20.09 ± 0.71 ng/mL) or 6-SG (20.30 ± 2.42 ng/mL) alone at the same concentration level (*p* < 0.001 vs. AN, *p* < 0.001 vs. 6-SG). Similarly, BA-SG [BA (12.5 µM) + 6-SG (12.5 µM), total concentration of 6.83 μg/mL] significantly reduced nitrite to 7.78 ± 3.16 ng/mL, which was significantly greater than BA (16.49 ± 2.09 ng/mL) or 6-SG (19.56 ± 3.14 ng/mL) (*p* < 0.0001 vs. LPS stimulation, *p* < 0.001 vs. BA, *p* < 0.0001 vs. 6-SG) (Figure 4B).

The stimulation of LPS also generated an excessive amount of nitrite in the lower compartment to 23.23 ± 1.40 ng/mL (*p* < 0.0001 vs. blank control: 0.24 ± 0.17 ng/mL, Figure 4A). The combination of AN-SG significantly lowered the level of nitrite to 15.16 ± 1.06 ng/mL (*p* < 0.0001 vs. LPS stimulation), and the single compounds, AN and 6-SG also reduced nitrite productions to 15.52 ± 0.71 ng/mL and 15.57 ± 0.29 ng/mL, respectively. However, the combined activities of AN-SG were not significantly higher than that of the single components. Similarly, BA-SG significantly reduced nitrite to 11.00 ± 3.86 ng/mL, which was significantly greater than BA (19.34 ± 1.09 ng/mL) or 6-SG (16.81 ± 2.44 ng/mL) (*p* < 0.0001 vs. LPS stimulation, *p* < 0.01 vs. BA, *p* < 0.05 vs. 6-SG, Figure 4B).

As shown in Figures 4C–F, LPS-stimulated cells produced significantly higher IL-6 (12.53 ± 3.70 ng/mL vs. 2.04 ± 0.11 ng/mL, *p* < 0.0001) and TNF-α (79.67 ± 0.10 ng/mL vs. 15.35 ± 0.01 ng/mL, *p* < 0.0001) in the upper compartment compared to that of the blank control. AN-SG significantly lower the IL-6 production (Figure 4C, 1.66 ± 0.62 ng/mL, *p* < 0.0001 vs. LPS) and TNF-α productions (Figure 4E, 20.40 ± 0.03 ng/mL, *p* < 0.0001 vs. LPS) in the upper compartments. Furthermore, the IL-6 reduction by AN-SG was greater than that of AN (4.86 ± 0.51 ng/mL, *p* < 0.0001) or 6-SG (3.40 ± 0.57, *p* < 0.01) alone (Figure 4C). Similarly, in the Figure 4E, the TNF-α reduction by AN-SG was greater than that of AN (37.36 ± 3.89 ng/mL, *p* < 0.0001) or 6-SG (38.13 ± 0.20 ng/mL, *p* < 0.0001), alone.

Similarly, in the tri-culture model, the nitrite, IL-6 and TNF-α inhibitory effects of BA-SG against LPS stimulation were compared to that of BA or 6-SG at 25 µM. The combination of BA-SG significantly lowered the IL-6 production (Figure 4D, 2.64 ± 0.70 ng/mL, *p* < 0.0001 vs. LPS) and TNF-α productions (Figure 4F, 29.00 ± 6.36 ng/mL, *p* < 0.0001 vs. LPS) in the upper compartments. However, there was no significant difference in the IL-6 reduction compared between BA-SG to BA (3.83 ± 0.52 ng/mL, *p* > 0.05) or 6-SG (2.63 ± 0.94 ng/mL, *p* > 0.05) in the Figure 4D. The TNF-α reduction by BA-SG was greater compared to BA (38.57 ± 0.56 ng/mL, *p* < 0.01) or 6-SG (38.13 ± 0.26 ng/mL, *p* < 0.01) either (Figure 4F). A similar trend of the IL-6 and TNF-α reduction was also found in the lower compartments, in which all the individual and combined activities showed significant and comparable effects.

#### 3.1.4 Enhanced effects of AN-SG and BA-SG in inhibiting NF-κB p65 translocation of N11 cells in the tri-culture model

The results of our previous study suggested that in the tri-culture model, the LPS-induced neuroinflammation was mediated by the NF-κB p65 translocation in the microglia cells (Zheng et al., 2021). In this study, the effects of AN-SG and BA-SG in inhibiting neuroinflammation on N11 cells in the tri-culture model was examined on the NF-κB p65 translocation by immunofluorescence staining.

**FIGURE 5:**
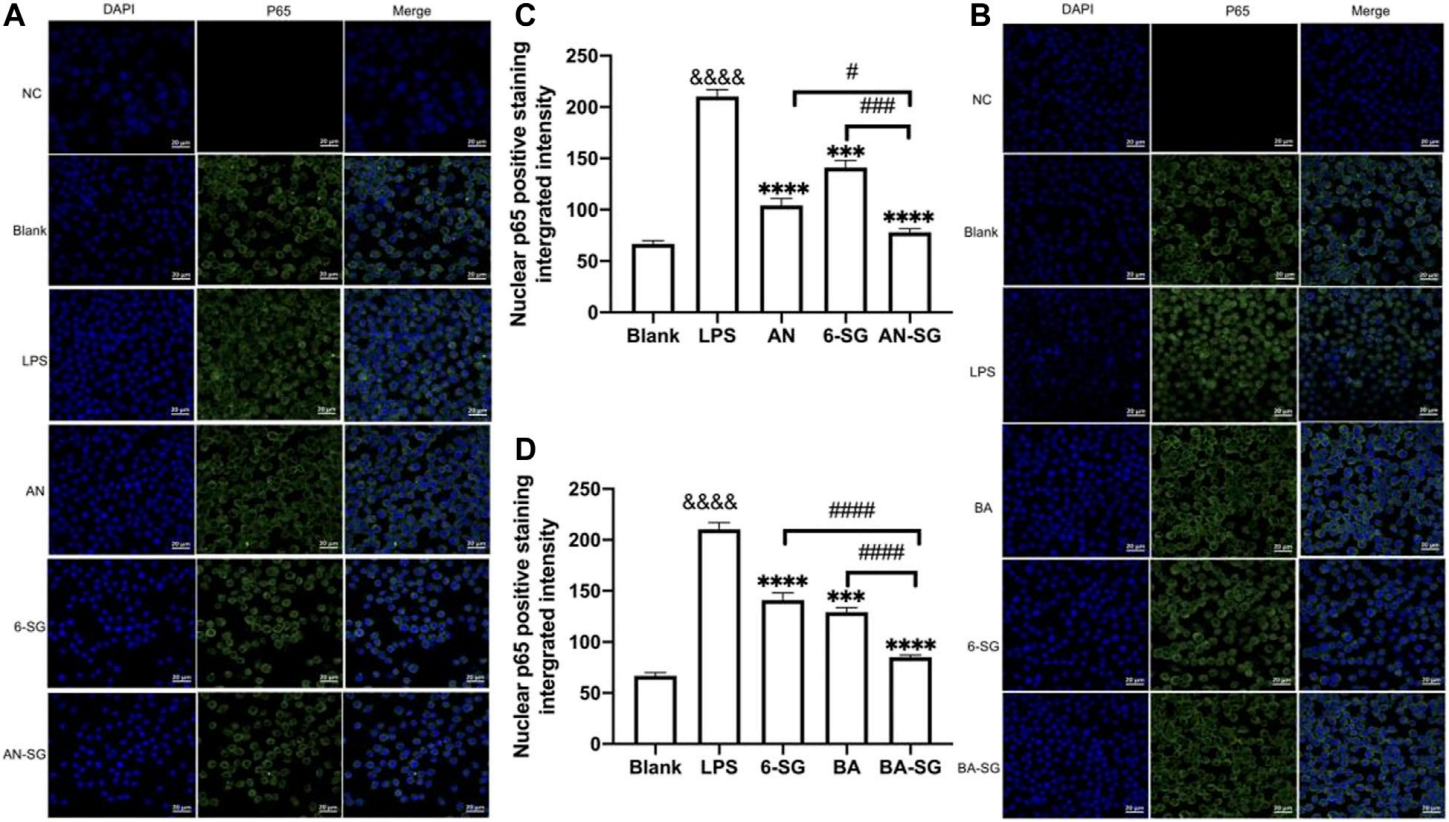
AN-SG and BA-SG exhibited greater effects in inhibiting LPS-induced NF-κB nuclear translocation than the individual component in the N11 cells of the tri-culture model. **(A)** AN-SG and **(B)** BA-SG for the immunofluorescence staining of NF-κB p65. Images were taken using a confocal microscope with × 63 magnification. Blue: DAPI in the nucleus, green: NF-κB p65 in the N11 cells. Scale bar = 20 μm. **(C, D)** Statistical analysis of the NF-κB p65 translocation activities by AN-SG and BA-SG using ImageJ. Data are shown as mean ± SEM (*n* > 3). &&&& *p* < 0.0001 vs. Blank, ***p < 0.001, ****p < 0.0001 vs. LPS, #*p* < 0.05, ###*p* < 0.001, ####*p* < 0.0001 vs. combination, by one-way ANOVA analysis and Tukey test for group comparison in GraphPad Prism 9.

As shown in Figure 5, the blank control showed that the NF-κB p65 (green fluorescence) was mostly expressed outside the nucleus (DAPI, blue fluorescence). In response to the LPS stimulation, increased expressions of NF-κB p65 were observed in the nucleus, as evidenced by the overlapping green in the blue fluorescence, suggesting that LPS triggered the increased translocation of NF-κB p65 to the cell nucleus after the 0.5 h’s incubation. All the individual and combined treatments appeared to inhibit the translocation to various degrees (Figures 5A,B). The statistical analysis (Figure 5C) demonstrated that AN-SG downregulated the integrated intensity of nuclear positive NF-κB p65 (77.96 ± 19.95, vs. LPS = 210.23 ± 28.00, *p* < 0.0001). The nuclear positive NF-κB p65 of AN-SG was significantly lower than that of AN (104.07 ± 24.78, *p* < 0.05) or 6-SG (140.93 ± 27.60, *p* < 0.001). BA-SG also significantly suppressed NF-κB p65 translocation (85.16 ± 8.91 vs. LPS = 210.23 ± 28.00, *p* < 0.0001), which the nuclear positive integrated intensity was significantly lower than that of BA (129.16 ± 19.34, *p* < 0.0001) or 6-SG (140.93 ± 27.60, *p* < 0.0001) alone (Figure 5D).

### 3.2 Enhanced effect of AN-SG and BA-SG in protecting endothelial tight junction of MVEC cells in the tri-culture system

**FIGURE 6:**
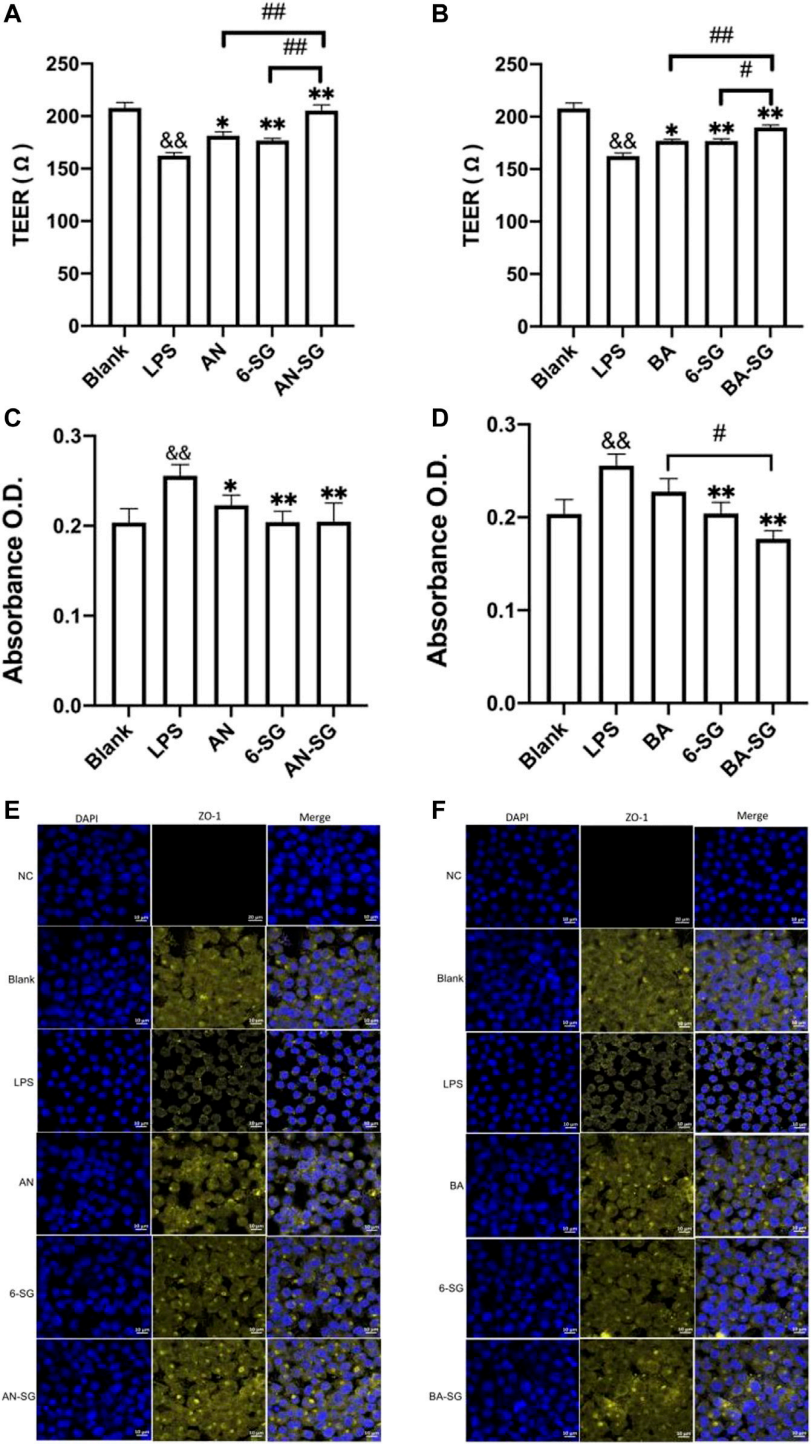
AN-SG and BA-SG demonstrated a more prominent effect in protecting the endothelial tight junction of MVEC cells in the tri-culture system against LPS-stimulated neuroinflammation. The measurements of TEER **(A)**, Evans blue absorbance **(C)** and tight junction protein ZO-1 **(E)** for AN-SG, and TEER **(B)**, Evans blue absorbance **(D)** and tight junction protein ZO-1 **(F)** for BA-SG in the LPS-activated tri-culture model. Immunofluorescence staining of ZO-1 in the MVEC cells of the tri-culture model. Images were taken using a confocal microscope with ×63 magnification. Blue: DAPI in the nucleus; yellow: ZO-1 in the microvascular endothelial cells. Scale bar = 10 μm && *p* < 0.01 vs. Blank, *p < 0.05, **p < 0.01 vs. LPS, #*p* < 0.05, ##*p* < 0.01 vs. combination, by one-way ANOVA analysis and Tukey test for group comparison in GraphPad Prism 9. O.D.: optical density.

In the MVEC cells of the tri-culture system, LPS exposure induced impaired endothelial tight junction as evidenced by a significant reduction of the TEER value in the LPS stimulated group (162 ± 12 Ω) compared with the blank control (207 ± 18 Ω, *p* < 0.01). As shown in Figure 6A, AN restored the TEER value to 181 ± 12 Ω (*p* < 0.05 vs. LPS), and 6-SG also increased the TEER value to 176 ± 6 Ω (*p* < 0.01 vs. LPS). However, AN-SG demonstrated the highest effect in restoring TEER value (205 ± 12 Ω, *p* < 0.01 vs. LPS), which was greater than that of AN and 6-SG alone (both *p* < 0.01).

The increased Evans blue absorbance also suggested the damaged endothelial tight junction upon the LPS simulation in the LPS group (0.28 ± 0.08) compared with the Blank group (Blank: 0.20 ± 0.06, *p* < 0.01, Figure 6C). Both AN and 6-SG alone significantly lowered the Evans blue absorbance to 0.22 ± 0.03 and 0.20 ± 0.04, respectively, *p* < 0.01 vs. LPS. AN-SG also lowered Evans blue absorbance (AN-SG: 0.21 ± 0.07, compared with LPS (*p* < 0.01). However, the combined treatment was not significantly stronger than the individual treatment.

In Figure 6B, both BA and 6-SG significantly increased the TEER value to 177 ± 5 Ω and 176 ± 6 Ω, respectively (*p* < 0.05 and *p* < 0.01 vs. LPS). BA-SG significantly restored TEER values (BA-SG: 190 ± 6 Ω, *p* < 0.01 vs. LPS) and showed greater ability than that of the BA (*p* < 0.01) and 6-SG (*p* < 0.05) alone. Similarly, BA-SG further lowered Evans blue absorbance (0.17 ± 0.02) than BA (0.22 ± 0.04) or 6-SG (0.20 ± 0.03) used alone (*p* < 0.05, Figure 6D).

The impaired tight junction in MVEC cells was further demonstrated by the immunofluorescence staining of ZO-1 upon the stimulation of LPS (Figures 6E, F). The tight junction protein ZO-1 in the blank tri-culture system was shown as a complete honeycomb structure, but the integrity was damaged after the LPS exposure for 24 h at 1 μg/mL. The single compounds AN, BA and 6-SG, and the AN-SG and BA-SG combinations increased the yellow honeycomb structure compared with the LPS and single component treatment. However, a prominent improved tight junction was seen after the treatment of AN-SG and BA-SG combinations. Compared with the AN and 6-SG used alone, AN-SG showed increased yellow honeycomb for the ZO-1 protein expression. Similarly, the BA-SG combination showed increased yellow honeycomb for the ZO-1 protein expression compared with the single compounds BA and 6-SG.

### 3.3 Enhanced effects of AN-SG and BA-SG in protecting neuronal survival and attenuating tau phosphorylation in the tri-culture model

#### 3.3.1 Enhanced effects of AN-SG and BA-SG in restoring neuronal survival of N2A cells

**FIGURE 7:**
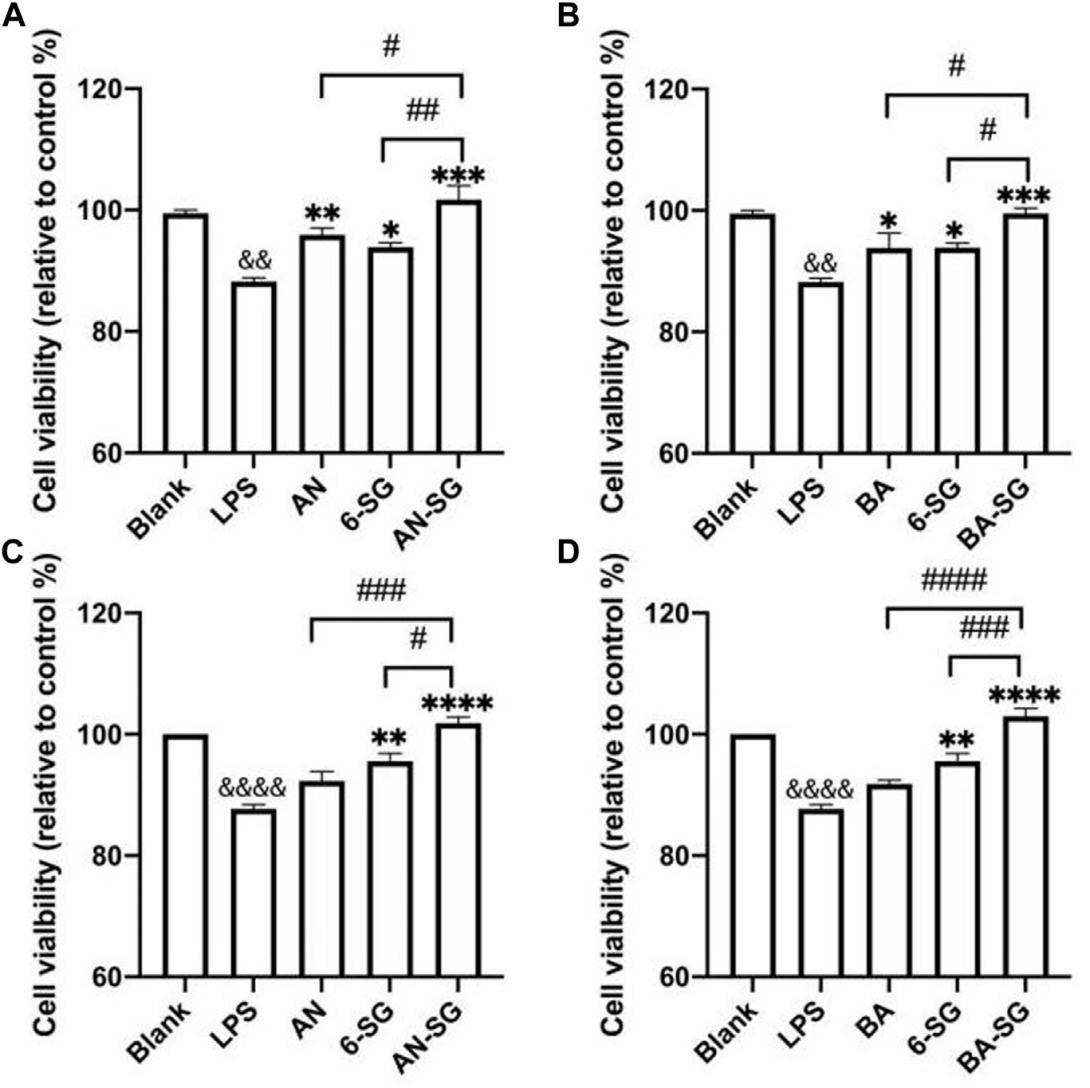
AN-SG and BA-SG exhibited greater restored cell viability in N2A cells of the tri-culture system against the LPS stimulation compared with their corresponding individual component. The Alamar blue assay was evaluated for **(A)** AN-SG and **(B)** BA-SG, and the MTT assay on **(C)** AN-SG and **(D)** BA-SG. Data are shown as mean ± SEM (*n* > 3). && *p* < 0.01, &&&& *p* < 0.0001 vs. Blank, *p < 0.05, **p < 0.01, ***p < 0.001, ****p < 0.0001 vs. LPS, #*p* < 0.05, ##*p* < 0.01, ###*p* < 0.001, ####*p* < 0.0001 vs. combination, by one-way ANOVA analysis and Tukey test for group comparison in GraphPad Prism 9.

The effects of AN, BA, 6-SG, AN-SG or BA-SG at 25 µM on cell viability of N2A cells in the tri-culture system was tested using Alamar blue and MTT assays. In Figure 7A, the Alamar blue assay demonstrated that the cell viability of N2A cells in the LPS group was significantly reduced compared with the blank group (88.23% ± 1.05% vs. Blank = 100%, *p* < 0.0001) after 24 h’ exposure. All treatments of AN (95.90% ± 2.20%, *p* < 0.01), 6-SG (93.89% ± 1.46%, *p* < 0.05) and AN-SG (101.71% ± 3.96%, *p* < 0.001) significantly restored the cell viability compared with that of LPS. In particular, AN-SG demonstrated the highest cell viability compared to AN (*p* < 0.05) or 6-SG (*p* < 0.01) alone. Similarly, in Figure 7B, all the single compounds BA (92.23% ± 4.22% vs. LPS, *p* < 0.05), 6-SG (94.12% ± 1.46% vs. LPS, *p* < 0.05) and combination BA-SG (100.21% ± 1.39% vs. LPS, *p* < 0.001) significantly restored the cell viability. BA-SG displayed the highest cell viability compared to BA or 6-SG (both *p* < 0.05).

MTT assay was conducted to confirm the data obtained from the Alamar blue assay, which showed a similar trend. In Figure 7C, LPS significantly decreased the cell viability compared with the blank group (87.74% ± 1.19% vs. Blank = 100%, *p* < 0.0001). AN (92.31% ± 2.68%, *p* > 0.05), 6-SG (95.55% ± 2.31%, *p* < 0.01) and AN-SG (101.83% ± 1.72%, *p* < 0.0001) significantly restored the cell viability compared with that of LPS. AN-SG demonstrated a higher cell viability level compared with AN (*p* < 0.01) or 6-SG (*p* < 0.05) alone. Similarly, in Figure 7D, BA-SG (102.97% ± 2.26% vs. LPS, *p* < 0.0001) also showed significantly enhanced cell viability, which was greater than that of BA (*p* < 0.0001) or 6-SG (*p* < 0.001) alone.

#### 3.3.2 Enhanced effects of AN-SG and BA-SG in reducing tau phosphorylation in N2A cells of tri-culture system

**FIGURE 8:**
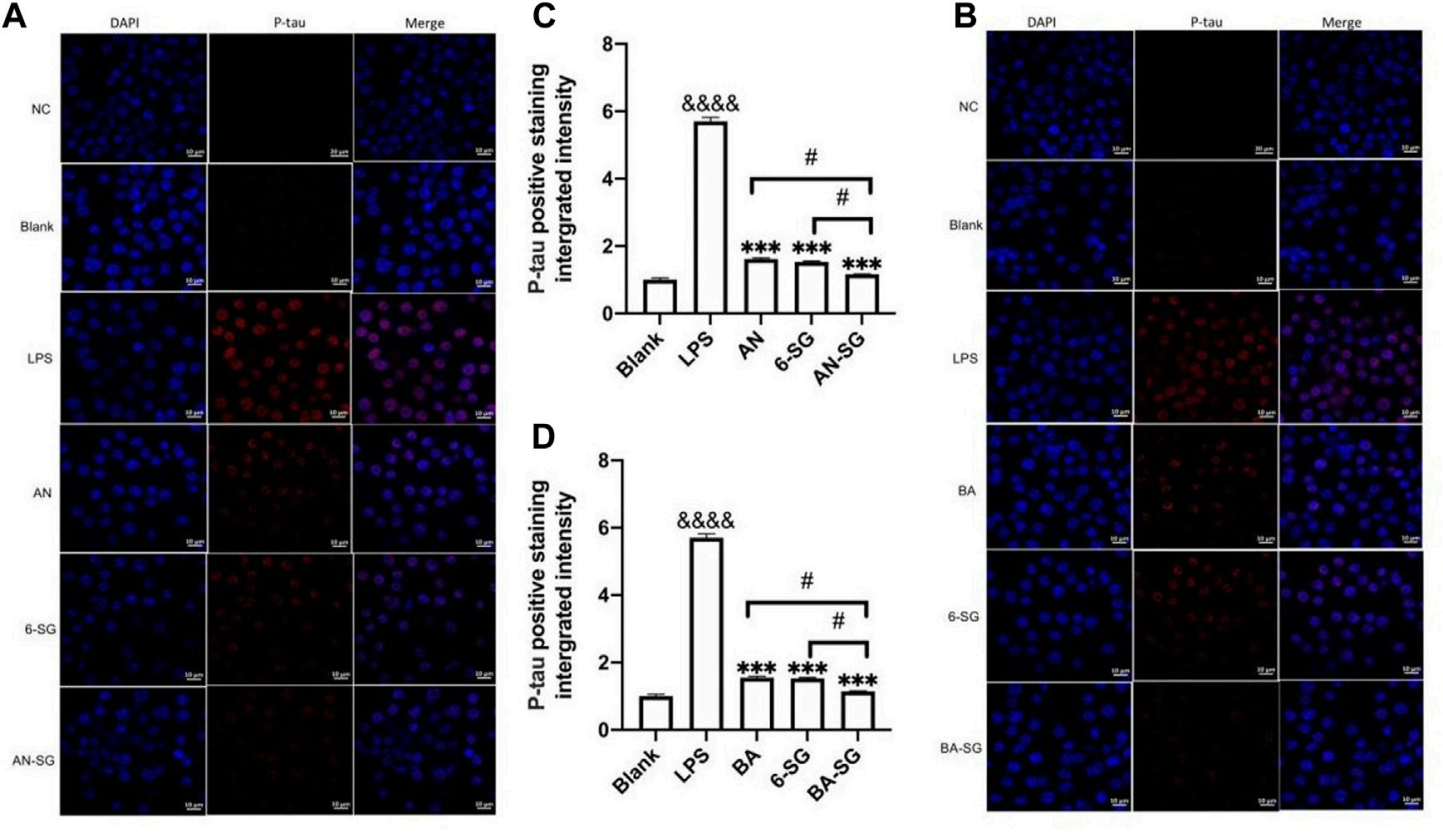
AN-SG and BA-SG exhibited greater p-tau reduction in N2A cells of the tri-culture system against the LPS stimulation compared with their corresponding individual component. Immunofluorescence staining of p-tau for **(A)** AN-SG and **(B)** BA-SG groups after the exposure of LPS for 0.5 h. Images were taken by a confocal microscope with × 63 magnification. Blue: DAPI in the nucleus. Red: p-tau in tri-cultured N2A cell lines. Scale bar = 10 μm. Use ImageJ to quantity the tau protein phosphorylation activity. **(C, D)** Statistical analysis of the p-tau expression by AN-SG and BA-SG using ImageJ. Data are shown as mean ± SEM (*n* > 3). &&&& *p* < 0.0001 vs. Blank, ***p < 0.001 vs. LPS, #*p* < 0.05 vs. combination, by one-way ANOVA analysis and Tukey test for group comparison in GraphPad Prism 9.

Immunofluorescent staining of p-tau protein in N2A cells was conducted to examine the capacity of AN-SG and BA-SG to attenuate p-tau expression in the LPS-induced neuroinflammation tri-culture system. As shown in Figure 8, the phosphorylation of tau protein (red fluorescence) increased significantly in the N2A cells after the LPS exposure for 0.5 h compared to that of the blank control group (Blank: 1.00 ± 0.08, LPS: 5.70 ± 0.20, *p* < 0.0001). AN, 6-SG and AN-SG appeared to decrease the tau phosphorylation compared with the LPS (Figure 8A). The statistical analysis (Figure 8C) demonstrated that all the treatments have significantly reduced the p-tau accumulation compared with LPS (*p* < 0.05). Particularly, the p-tau accumulation in AN-SG (1.16 ± 0.01) was significantly lower than that of AN (1.62 ± 0.05) or 6-SG (1.53 ± 0.04) individually (*p* < 0.05, respectively).

Similarly, it was obvious that BA, 6-SG and BA-SG decreased tau phosphorylation compared with the LPS treatment (BA: 1.55 ± 0.06, 6-SG: 1.53 ± 0.04, BA-SG: 1.14 ± 0.02, *p* < 0.001, respectively, Figures 8B, D). However, BA-SG demonstrated a more effective p-tau reduction compared with the BA or 6-SG alone (*p* < 0.05, respectively).

## 4 Discussions

This study investigated the anti-neuroinflammatory activities of two combinations, AN-SG and BA-SG, in comparison to that of their individual components in the LPS-stimulated neuroinflammation tri-culture model. Our results demonstrated that these combinations exhibited pronounced effects in suppressing neuroinflammation mediated by N11 microglia cells and, in turn, protecting endothelial tight junction and restoring neuronal cell viability. Noticeably, the combined effects were generally greater than the single component used alone.

Neuroinflammation is a complex inflammatory process in the CNS that plays a vital defensive role against numerous pathogens, toxins, or factors that produce neurodegeneration. Recent research suggested that dysfunctional cell-cell signaling in the NVU is a defining feature of CNS illnesses (Zlokovic, 2005; Benarroch, 2007; Zarekiani et al., 2022). The CNS’s structural and functional integrity is dependent on the function of NVU, which regulates transport across the BBB (Erickson et al., 2020; Pluimer et al., 2020; Salmina et al., 2021). Increasing evidence showed that disruption of functional interactions among the cells in NVU is the early hallmark of Alzheimer’s disease (Montagne et al., 2017; Liebner et al., 2018; Yu et al., 2020).

Our results suggested that the combinations of AN-SG and BA-SG significantly inhibited NO, IL-6, and TNF-α in mono-cultured N11 and the tri-culture cells. Microglia N11 cells are the resident innate immune cells in the NVU and produce several proinflammatory factors (IL-1, TNF-α, NO, PGE2, superoxide) within the brain (Block and Hong, 2005). Among these, high NO levels can increase neuroinflammation leading to tissue damage and neuronal death (Liy et al., 2021). IL-6 is a pleiotropic cytokine that participates in a variety of pathways, including immunological responses, inflammation, hematopoiesis, bone metabolism, and embryonic development (Tanaka et al., 2014). TNF-α is an inflammatory cytokine that promotes apoptosis and activates NF-κB, resulting in the generation of proinflammatory cytokines and glial activation, which leads to neuroinflammation and neuronal death (Jung et al., 2019). NF-κB is the key transcription factor that plays a central role in the neuroinflammatory pathways. It is a crucial regulator of cellular gene transcription that can regulate a variety of cytokines and receptors, such as NO, IL-6 and TNF-α (Zhang and Sun, 2015).

In this study, the single herbal compounds and their combinations effectively inhibited the NF-κB p65 translocation, which partly explains the mechanisms underlying their anti-neuroinflammatory activities. In particular, our results showed that the AN-SG and BA-SG combinations produced greater effects on many neuroinflammatory mediators, including nitrite, IL-6 and TNF-α compared with the single components. To the best of our knowledge, this is the first study that demonstrated the combined effects of AN and 6-SG for anti-neuroinflammatory activity. Our results of the BA-SG combination are somewhat in line with the findings from Suzuki et al., where a combination of aqueous extract of *Scutellaria* root (BA is a main bioactive ingredient) and 6-SG was shown to produce an advanced effect on cyclic adenosine monophosphate phosphodiesterase inhibitory activity (Suzuki et al., 1991), which may contribute to the attenuated neuroinflammation (Chuang et al., 2017).

In the NVU, astrocytes, microglia and pericytes are directly localized around the endothelial cells to aid the connection between blood supply and metabolic needs and also release several substances that improve and preserve the BBB integrity (Ronaldson and Davis, 2012). Excessive activation of microglia can have a negative impact on the BBB function. In particular, the excessive release of NO and IL-6 from the activated microglia has been demonstrated to impair the BBB’s permeability, enabling potentially toxic chemicals to actively enter the brain (Thiel and Audus, 2001; Mayhan, 2002; Takeshita et al., 2021). The downregulation of paracellular tight-junction proteins such as claudin-5 (CLDN5), occluding, and ZO-1 by reactive microglia and astrocytes are believed to contribute to the leaky BBB (Morris et al., 2018). Numerous studies demonstrated that increased BBB permeability is observed in a variety of neurological and psychiatric disorders, including stroke, epilepsy, amyotrophic lateral sclerosis, Alzheimer’s disease and Parkinson’s disease, implying that the compromised BBB plays a role in the pathogenesis and/or severity of brain diseases (Liu et al., 2015; Patel and Frey, 2015; Małkiewicz et al., 2019; Hussain et al., 2021). Thus, therapeutic targets that could minimize the infiltration and invasion of peripheral chemicals or pathogens into the CNS or reduce damage to the NVU that protect the BBB, hold a high promise for treating chronic neurological illnesses and acute CNS traumas. Our present study showed that AN-SG and BA-SG inhibited the inflammatory mediators of NO, IL-6 and TNF-α, which in turn, led to reduced permeability and restored endothelial tight junction, suggesting their the BBB protective effects against cytokine-mediated damage from microglial cells (Haruwaka et al., 2019). This is consistent with the previous studies where AN and BA were shown to protect against BBB damage induced by the production of pro-inflammatory cytokines, and increase ZO-1 expression to reduce BBB permeability (Zhu et al., 2012; Wang et al., 2022).

Abnormal tau phosphorylation accumulation is a distinguishing characteristic of some neurodegenerative diseases, such as Alzheimer’s disease (Wolfe, 2012). Tau proteins are often seen as microtubule-associated proteins in the brain. They are widely distributed throughout the neurons of the CNS and play a large part in preserving the stability of microtubules in axons (Buée et al., 2000). Abnormal phosphorylation of tau proteins (p-tau) results in impaired microtubule stability and the production of potentially neurotoxic aggregates (Johnson and Stoothoff, 2004). An increasing number of studies indicate that the sustained neuroinflammatory process involving microglia and astrocytes activation significantly contributes to the progression of tau pathology and neurodegenerative diseases, emphasizing the significance of neuroinflammation as a therapeutic target for neurodegenerative diseases (Laurent et al., 2018). In addition, inhibiting microglial inflammatory mediator production was shown to protect neurons and reduce p-tau in the tau transgenic mouse model. Previous animal studies have demonstrated that AN and BA used individually, can reduce p-tau levels (Serrano et al., 2014; Asai et al., 2015; Gu et al., 2016; Patel et al., 2021; Sonawane et al., 2021). The mechanism of action of AN and BA were both related to the activation of the Nrf2-mediated p62 signaling pathway (Serrano et al., 2014; Gu et al., 2018; Sonawane et al., 2021; Shengkai and Yazhen, 2022). However, the evidence for the potential effect of 6-SG to inhibit p-tau level is lacking. Thus, the strengthened p-tau inhibition by AN-SG maybe is predominately caused by AN, which is potentiated by 6-SG. However, if the Nrf2-mediated p62 signaling pathway plays a role in the combined action warrants further investigation. Overall, our results suggested that AN-SG and BA-SG reduced cytokines such as NO, IL-6, and TNF-α, which can protect the BBB tight junction and the neuron survival and lower the p-tau level. These results provide evidence that AN-SG and BA-SG could provide potential benefits for the treatment of neurodegenerative diseases such as Alzheimer’s disease, Parkinson’s disease and multiple sclerosis (Laurent et al., 2018; Singh et al., 2020; Shengkai and Yazhen, 2022). Further study to explore the effects of these combinations on other Alzheimer’s disease-relevant biomarkers, such as Amyloid beta, which is an early event associated with the onset and progression of neurodegeneration (Roy et al., 2020), is warranted.

Previously, we have established the LPS-stimulated neuroinflammation tri-culture model as a quick and practical tool for quick drug-screening targeting neuroinflammation (Zheng et al., 2021). This model was successfully applied in the current study where promising anti-neuroinflammatory, endothelial tight junction protective and neuroprotective effects of three herbal bioactive components and their combinations were demonstrated. However, there are several limitations of this study. Firstly, the study focused on the NF-κB nuclear translation pathway as an associated mechanism of AN-SG and BA-SG against neuroinflammation. Other pathways or key gene targets that play major roles in modulating microglia-induced neuroinflammation, such as Nrf-2/HO-1, nucleotide-binding oligomerization domain-like receptor protein three inflammasome, and mitogen-activated protein kinase pathways, were not investigated (Bernhardi, 2009; Song et al., 2017; Chen et al., 2020; Upadhayay and Mehan, 2021). Secondly, the combinations demonstrated an enhanced effect on the biomarkers related to neuroinflammation, endothelial tight junction and neuronal survival in the *in vitro* tri-culture system; these effects were not validated in the whole organism. Thirdly, the effects of the herbal components and their combinations were investigated at only one concentration (25 µM) in this study. Lastly, since synergy quantified needs have a range of doses to get linear dose–effect curves (Foucquier and Guedj, 2015), the consequences can only be described as an enhanced effect. Nonetheless, the enhanced effects of the two herbal combinations against neuroinflammation in our study provide valuable evidence to warrant further investigation of these combinations in the animal and human studies as potential therapy options for neurodegenerative diseases.

## 5 Conclusion

The present study evaluated the anti-neuroinflammatory effects of AN, BA, 6-SG and two of their combinations, AN-SG and BA-SG in a tri-culture model. Our findings revealed that AN-SG and BA-SG exhibited greater anti-neuroinflammatory activities in the microglia cells compared to their individual components, and in turn produced a greater endothelial tight junction protection and improved neuronal survival. Future study is warranted to further explore the potential of AN-SG and BA-SG as anti-neuroinflammatory and neuroprotective agents for neurodegenerative diseases in animal and clinical studies.

## Data Availability

The raw data supporting the conclusion of this article will be made available by the authors, without undue reservation.
