# Supplementary material for: Natural product-based bioactive agents in combination attenuate neuroinflammation in a tri-culture model
Source: Front Pharmacol. 2023 Feb 15;14:1135934. doi: 10.3389/fphar.2023.1135934 (PMC9979791; doi:10.3389/fphar.2023.1135934)
Supplement: Supplementary file 1 [file DataSheet1.pdf]

frontiers

Supplementary Material

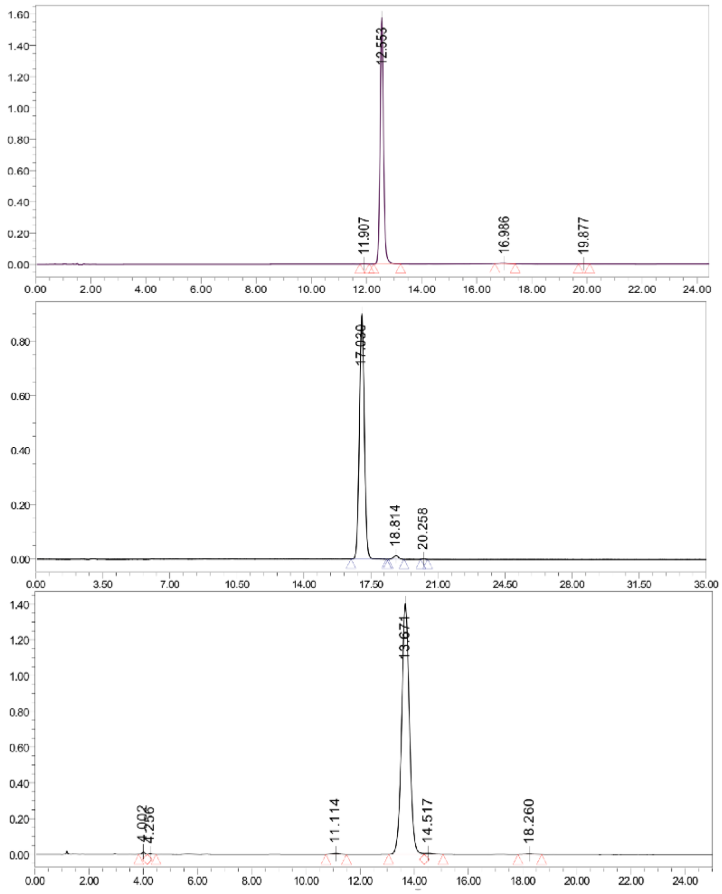

A

Peaks	Retention (min)	Peak area (mAU*s)	Peak height (µV)	Peak are (%)
1	11.907	15070		0.11
2	12.553	14016843		99.33
3	16.986	65681		0.47
4	19.877	14248		0.1

B

Peaks	Retention (min)	Peak area (mAU*s)	Peak height (µV)	Peak are (%)
1	4.732	9788	0.09	1624
2	5.898	22093	0.21	3453
3	12.981	13427	0.13	984
4	13.651	10351271	98.63	1052992
5	21.685	8895	0.08	620
6	22.593	15742	0.15	1076
7	24.334	73520	0.7	4204

C

Peaks	Retention (min)	Peak area (mAU*s)	Peak height (µV)	Peak are (%)
1	4.002	84421	14096	0.3
2	4.256	25213	0.09	3935
3	11.114	96920	0.34	5031
4	13.671	27854508	98.7	1401299
5	14.517	108216	0.38	5293
6	18.26	52082	0.18	2143

Supplementary Figure 1. HPLC analysis of pure reference compound, BA, AN and 6-SG, used in the study (Chengdu BioPurify Pty Co., China). (A) BA purity: 98.63%, retention time: 11.91 min, (B) AN purity: 99.33%, retention time: 4.73 min, (C) 6-SG purity: 98.70%, retention time: 4.00 min.
